# Physiology-Mimicking Microfluidic Oxygenator with Good Hemocompatibility for *In Vitro* Respiratory Support of Preterm Infants

**DOI:** 10.3390/mi17060745

**Published:** 2026-06-20

**Authors:** Yu Tao, Yao Lu, Weijun Zeng, Donggen Xiao, Haixuan Sun

**Affiliations:** 1 School of Biomedical Engineering (Suzhou), University of Science and Technology of China, Hefei 230026, China; 2 Suzhou Institute of Biomedical Engineering and Technology, Chinese Academy of Sciences, Suzhou 215163, China

**Keywords:** microfluidics oxygenators, preterm infants, extracorporeal respiratory support, hemocompatibility

## Abstract

Preterm infants, especially extremely preterm infants under 28 weeks of gestation, face high mortality rates due to respiratory distress resulting from pulmonary immaturity. Conventional mechanical ventilation and extracorporeal membrane oxygenation (ECMO) therapy inevitably cause irreversible lung injury or severe complications, respectively. Here, we developed a microfluidic oxygenator (MO) mimicking the human alveolar-capillary barrier to provide respiratory support for preterm infants. These structures promoted uniform flow distribution, reduced high-shear stress and flow stagnation, and improved gas exchange efficiency. In vitro experiments demonstrated that a single-layer MO raised blood oxygen saturation from 64.7% to 96.5% at 8 mL/min, with a corrected vol% oxygen transfer of 5.24% (52.4 mL O_2_/L blood). Hemolysis and coagulation measurements after a 6 h circulation confirmed good hemocompatibility, with most blood damage attributable to the pump. An eight-layer stacked MO was configured with a total priming volume of approximately 5.6 mL and a pressure drop of 25–35 mmHg at 24–40 mL/min, indicating its potential in pumpless extracorporeal circulation for preterm neonates. This MO holds promise for providing minimally invasive and customizable respiratory support in an artificial uterus system.

## 1. Introduction

In global neonatal healthcare, approximately 15 million preterm births occur each year, accounting for more than one-tenth of all neonates. Among these preterm infants, about 1 million infants die annually due to complications of prematurity, constituting nearly one-fifth of all deaths in children under the age of five [1]. The severity of complications is inversely correlated with gestational age at birth [2]. Although advances in neonatal intensive care have improved outcomes for preterm infants, extreme prematurity remains a condition of significant clinical severity, characterized by substantial risks of complications and death from pulmonary immaturity [2,3,4,5,6].

Lung-protective continuous positive airway pressure (CPAP) is a primary therapeutic approach for respiratory support of babies in clinical practice [7]. However, CPAP has the risk of causing lung injury and complications of the circulatory system in these infants [8], including diminished pulmonary blood flow, impaired cardiac function, and reduced cardiac output [9,10,11,12], which would increase cardiac workload for neonates [13]. Extracorporeal membrane oxygenation (ECMO), commonly known as “artificial lung”, is an alternative treatment applicable to neonates, children, and adults with respiratory failure. Nevertheless, due to its large priming volume (38 mL for QUADROX-i Neonatal [14,15], and 43 mL for CAPIOX^®^ FX05 [16]), this technology is not suitable for extracorporeal respiratory support in premature infants. Moreover, there may be high-risk complications associated with the treatment, such as systemic coagulation disorders and inflammatory responses [17,18,19,20]. The primary factor compromising the hemocompatibility comes from the mechanical damage by the centrifugal pump, and non-physiological blood flow patterns in some hollow fiber-based oxygenators, including regions of elevated shear stress, blood stasis, and abrupt transitional flow zones [21,22,23]. In addition, the artificial blood-contacting surfaces of the entire ECMO circuit—such as tubing, connectors, and the oxygenator membrane—can themselves initiate surface-induced blood activation, leading to coagulation and complement-mediated inflammation [24]. Connectors, in particular, have been identified as a major contributor to thrombosis due to disturbed flow, recirculation zones, and elevated shear stress gradients [25]. Therefore, developing an efficient blood oxygenator that mimics physiological characteristics is essential to both minimize adverse flow phenomena and reduce surface-induced activation, thereby improving survival outcomes for preterm infants.

Currently, microfluidic technology has been applied to the development of blood oxygenators attributing to the biocompatibility and flexibility [26]. Compared to hollow-fiber oxygenators, microfluidic oxygenators feature thinner gas-permeable membranes (30–50 μm) and channel heights (20–200 μm) [27,28,29], which significantly reduce the oxygen diffusion distance, thereby improving the gas transfer efficiency [30,31,32]. By utilizing multi-level channel structures fabricated by microfabrication technology to mimic the pulmonary vascular network, hemocompatibility is expected to be improved in extracorporeal oxygenation devices [33]. For instance, Dabaghi et al. constructed a dual-side gas transfer microfluidic oxygenator using a polydimethylsiloxane (PDMS) membrane reinforced with an ultrathin stainless-steel mesh, achieving superior oxygen transfer performance (100% saturation) at a flow rate up to 30 mL/min [27], whereas the fabrication process is relatively complex. Santos et al. fabricated a microfluidic oxygenator with varying channel height in the blood layer by machining to better mimic the diameter ratios of human blood vessels; however, this oxygenator exhibits a relatively large pressure drop [34]. Further, a clinical-scale microfluidic oxygenator fabricated with high-precision processes successfully completed a 24 h large-animal trial at a blood flow rate of 750 mL/min. However, visible thrombi formed in channels (2–19%) after a 24 h operation [35]. Since external pumps used in the extracorporeal support system generate mechanical shear forces on blood that can cause hemolysis and increase cardiac workload [36], pumpless oxygenators have been proposed [29]. In these devices, blood is driven by the mean arteriovenous pressure difference (MAPD) which typically ranges from 20 to 60 mmHg in adults [37]. Additionally, the scalability of microfluidic oxygenators enables them to adjust the blood flow rate and priming volume, making it more suitable for preterm neonates of varying gestation (i.e., different weights) to provide lung assist function. While previously designed microfluidic oxygenators show promising potential, they have limitations, such as inadequate utilization of the oxygenation area due to structure, low hemocompatibility, and mismatched flow rates in preterm infants.

Here, we proposed a novel design and fabrication method for PDMS-made microfluidic oxygenators (MOs) that mimic the vessel diameter ratios and branching angles of human vascular network. The blood channel layer was designed based on Murray’s law [38,39], achieving a priming volume of only 0.72 mL for a single-layer MO—a significant reduction compared to hollow-fiber blood oxygenators. This device is intended for preterm neonates with birth weights below 1280 g, whose typical blood flow rate is 64 mL/min (approximately 50 mL/kg/min) [29]. The target blood flow rate for single MO is 3–8 mL/min. The total blood flow rates can be increased by connecting multiple oxygenator units in parallel, allowing the device to meet the required blood flow range for such preterm infants. An in vitro circulation system was established to evaluate the pressure drop and blood damage. The single MO device developed in this work demonstrated an increase in oxygen saturation from 64.7% to 96.5% at a blood flow rate of 8 mL/min. Compared to reported microfluidic blood oxygenators, this device significantly improves oxygenation efficiency.

## 2. Materials and Methods

### 2.1. Oxygen Transfer Model

**Figure 1:**
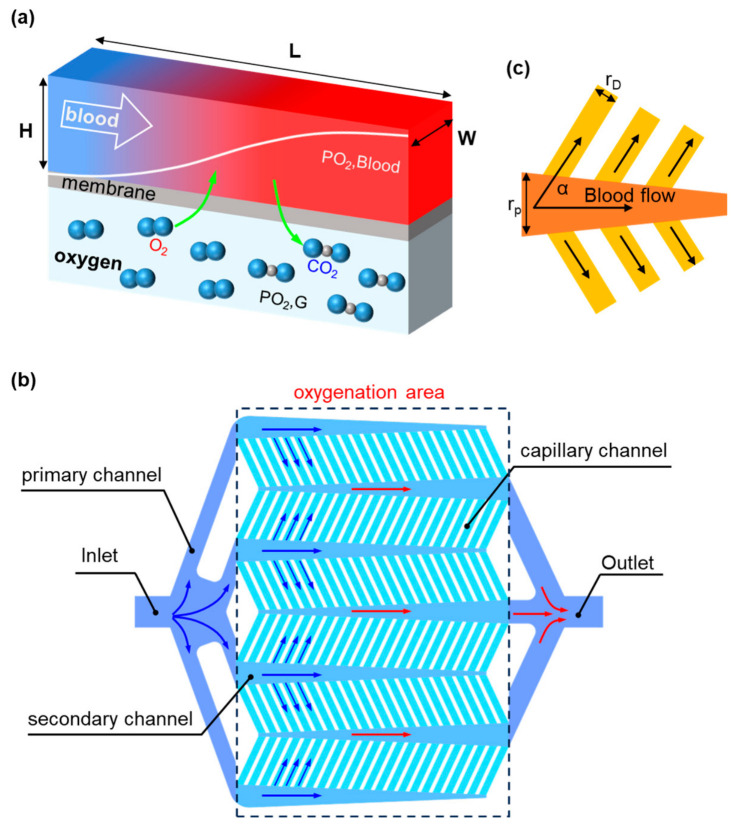
Design principle of the microfluidic oxygenator (MO) device. (**a**) A simplified model of the human alveolar-capillary physiological barrier for the microfluidic oxygenator. (**b**) Top view diagram of the main branching structure based on Murray’s law. (**c**) Top view of the basic blood flow network with multi-level channels.

Figure 1a illustrates a simplified blood oxygenation model established by simulating the physiological structure at the alveolar-capillary barrier, showing its trilaminar architecture comprising a blood channel layer, a permeable membrane, and a gas channel layer. Gas exchange in blood follows passive diffusion driven by gas partial pressure gradients, with gas spontaneously diffusing across the biological membrane from high to low partial pressure. In the designed trilaminar structure, the gas channel maintains a high oxygen partial pressure (PO_2_) and low carbon dioxide partial pressure (PCO_2_) level. When the blood PO_2_ is lower than that in the gas channel, oxygen molecules continuously diffuse from the gas side into the bloodstream. Similarly, carbon dioxide diffuses down its partial pressure gradient from the blood into the gas layer. Given that oxygen and carbon dioxide transport occur concurrently, the present oxygenation model primarily focuses on the oxygen transfer behavior. Reducing the membrane thickness has been consistently identified as a key determinant for enhancing the gas permeability, as it directly minimizes mass transfer resistance [40,41]. In this model, we adopted some physically justified simplifications and assumptions [41]: (1) The microchannels in the blood layer are rectangular with a defined length (L), height (H), and width (W). (2) Oxygen diffusion is simplified as a one-dimensional transport process perpendicular to the permeable membrane surface. (3) Blood is treated as a homogeneous fluid characterized by effective oxygen solubility and diffusivity. (4) The thickness of the mass transfer boundary layer in the blood channel is approximately half of the channel height. (5) The oxygen partial pressure in the gas channel is uniform and constant, and there is no significant variation along the channel height. (6) The oxygen partial pressure in the blood channel varies exclusively along the flow direction. These assumptions provide the basis for deriving the analytical solutions of oxygen saturation along the channel length, which have been validated as a reasonable approximation [42,43,44].

### 2.2. Structural Design of the Blood Layer

To achieve hemocompatibility and efficient oxygenation in the MO device, we divided the blood channels into three levels based on the geometry relationship of the human capillary network, including primary channels, secondary channels and capillary channels, as shown in Figure 1b. The blood flows in the direction indicated by the arrows, and the oxygenation process is designed to occur in the capillary channels. Since flow resistance and branching angles are the same across channels at the same level, blood can theoretically form a uniform flow pattern within the multi-level channel network [39,45]. Oxygenation efficiency is a key performance metric for the microfluidic blood oxygenator, primarily dominated by the geometric parameters of its capillary channels—especially length (*L*), height (*H*), width (*W*), and total gas exchange area (*A*) [28]. These dimensions are determined by the thickness and permeability of membrane, the pressure drop, the blood flow rate, and the oxygen partial pressure gradient across the membrane. The oxygenation target for a single-layer MO was set to increase the blood oxygen saturation from 65% to 95% at a target blood flow rate of 5 mL/min [40].

(1) Capillary channel

A is determined by the blood flow rate (*Q*), effective solubility of oxygen in blood ($S_{B,O_{2}}$), effective diffusion resistance ($R_{D,O_{2}}$), the oxygen partial pressure in the gas (*PO_2,G_*) and blood (inlet *PO_2,B,i_* and outlet *PO_2,B,o_*), as given by [40,41]:

(1) $$A=Q\times S_{B,O_{2}}\times R_{D,O_{2}}\times \ln\left(\frac{{PO}_{2,B,i}-{PO}_{2,G}}{{PO}_{2,B,o}-{PO}_{2,G}}\right)$$

*PO_2,B,i_* and *PO_2,B,o_* can be derived from the corresponding blood oxygen saturation levels using the Hill equation [46]. Generally, the blood oxygen saturation at the inlet and outlet of oxygenator are 65% and 95%, respectively, with corresponding oxygen partial pressures at 33.4 mmHg and 100 mmHg. In addition, the effective diffusion resistance encountered by oxygen as it travels across the blood-gas barrier is given by [41]:

(2) $$R_{D,O_{2}}=\frac{\delta_{M}}{P_{M,O_{2}}}+\frac{H/2}{S_{B,O_{2}}\times D_{B,O_{2}}}$$

where *δ_M_* and $P_{M,O_{2}}$ are the thickness and the oxygen permeability of the membrane, respectively. The diffusion coefficient of oxygen in blood is denoted as $D_{B,O_{2}}$_._ The optimal PDMS membranes were selected based on the thickness, permeability and mechanical properties (Table S1). In this work, we selected a 20-μm-thick PDMS membrane with a $P_{M,O_{2}}$ of 4.35 × 10^−9^ mL O_2_/cm/s/mmHg. $S_{B,O_{2}}$ and $D_{B,O_{2}}$ are 8.99 × 10^−4^ mL O_2_/mL blood/mmHg and 1.27 × 10^−6^ cm^2^/s, respectively [41]. To improve fabrication precision and gas layer utilization, a 6-inch silicon wafer was used to fabric the gas layer mold. The estimated A was 0.0030 m^2^ after deducting the non-oxygenating membrane area [45], which yielded a preliminary $R_{D,O_{2}}$ according to Equation (1). Applying this $R_{D,O_{2}}$ to Equation (2) and making appropriate adjustments, the channel height (H) was determined to be 80 μm, corresponding to an adjusted A of 0.0028 m^2^ and an $R_{D,O_{2}}$ of 3.98 × 10^6^ mmHg·s/mL O_2_. Note that the model only accounts for the oxygenation within the capillary channels. In our fabricated oxygenator, nearly all blood channels are covered by the gas layer, which may also contribute to oxygen transfer in primary and secondary channels. Thus, the model provides a conservative estimate, and the actual device may achieve higher oxygenation efficiency.

(2) Pressure drop

The pressure drop Δ*P* across the capillary channel network within the blood layer can be derived from the Hagen–Poiseuille equation [37]:

(3) $$∆P=\frac{12\times \mu \times L\times Q}{W\times H^{3}\times \left(1-\frac{0.63\times H}{W}\right)}$$

where *μ* is the viscosity of blood. To enable a pumpless circulation, the pressure drop across the MO and other circuit elements should be maintained within the range of MAPD of preterm infants (35–45 mmHg) [29,47]. Given the required oxygen exchange area *A = L × W* and the target pressure drop Δ*P*, *L* and *W* were calculated to be 1.35 cm and 0.2 cm, respectively, by solving Equations (1) and (3) simultaneously.

(3) Secondary channels

The dimensions and bifurcation angle of the blood channels are the key bionic features that determine blood flow patterns. We followed Murray’s law to establish the relationship between vessel diameter and blood flow. It states that the cube of the radius of a parent vessel equals the sum of the cubes of the radii of its daughter vessels. Also, a relationship between the bifurcation angle (*α*) and diameters of the parent and daughter vessels are given by [38,48]:

(4) $$\cos\left(\alpha \right)=\frac{r_{p}^{2}-{\left(r_{p}^{3}-2\times r_{D}^{3}\right)}^{\frac{2}{3}}}{2\times r_{D}^{2}}$$

where *r_p_* and *r_D_* denote the hydraulic radii of the parent channel and the daughter channel, respectively. For the rectangular microchannels in microfluidic devices, the equivalent radius can be calculated by *r = W·H*/(*W + H*) [38]. Applying this calculation methodology, the average bifurcation angle between the secondary channels and the capillary channels was determined to be 65° (as shown in Figure 1c). To achieve a spatially ordered and efficient layout of the vascular network within the blood layer, the dimensions of the secondary channels were designed as linear variations based on Murray’s law; specifically, the width increases or decreases linearly from 0.8 mm to 6 mm, and height increases or decreases linearly from 110 μm to 200 μm. Further, the heights of the inlet and outlet areas were set to 400 μm and 300 μm, respectively, to ensure stable blood flow and minimize pressure drop.

### 2.3. Design of the Gas Layer

The gas layer has a relatively minor impact on the overall device performance, provided it allows unobstructed gas supply to the membrane [40]. Accordingly, the gas layer was designed to meet the following objectives: (1) fully cover the capillary channel area to ensure sufficient oxygen exchange; (2) employ a mechanically stable support structure to prevent membrane collapse under lateral flow pressure; and (3) ensure uniform and stable gas distribution across the exchange area. The implemented design for the gas layer featured an array of micropillars with diameters of 100 μm, heights of 150 μm and center spacings of 200 μm on an area of 0.0119 m^2^. The gas cavity with densely arrayed micropillars (~25,000) forms a pulmonary alveolus-like structure, providing an effective gas exchange area of 0.0042 m^2^ for a single-layer MO, accounting for 75% of the total blood channel layer.

### 2.4. MO Fabrication

The blood and gas layers of the MO devices were fabricated through PDMS replica molding, which requires corresponding negative molds. The mold for the blood layer was prepared from aluminum via computer numerical control (CNC) machining (Beijing Jingdiao Group Co., Ltd., Beijing, China), which enables the accurate fabrication of subtle height gradients on the mold surface. Surface roughness of the finished mold measured with a diamond-tip stylus profilometer (MMD-PG100, Wilson Precision Instruments, Xi′an, China) showed a maximum of about 160 nm, which is suitable for subsequent PDMS bonding. Given the small size and dense arrangement of the micropillars on the gas layer, the mold of the gas layer was made using SU-8 photoresist (SU-8 2150, Micro-chem Corp, Westborough, MA, USA) by photolithography (Xi’an Boyan Micro-nano Informational Technology Co., Ltd., Xi’an, China).

**Figure 2:**
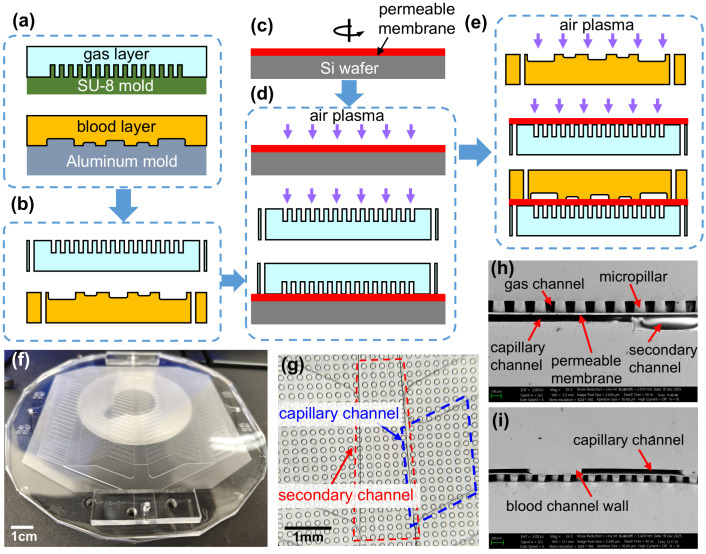
Fabrication process of the MO device. (**a**) PDMS was cast onto the molds, degassed, cured, and peeled off to form the blood and gas layers. (**b**) Inlets and outlets were drilled using a biopsy punch. (**c**) Preparation of permeable membrane by spin-coating PDMS on a Si wafer. (**d**) The gas layer was bonded to the PDMS membrane via air plasma activation. (**e**) The blood layer was aligned and bonded to the opposite side of the PDMS membrane already attached to the gas layer. (**f**) Image of the single-layer MO device. (**g**) Top-view optical micrograph (4×) of the fabricated MO, showing the micropillars (diameter: 100 μm) of the gas layer and the secondary channel and capillary channels. (**h**,**i**) Scanning electron microscope (SEM) images showing the trilayer structure of the MO.

The fabrication process for the MO device is described in Figure 2. Briefly, a mixed PDMS solution (base:curing agent = 10:1) was poured onto the prepared mold, degassed and cured in an oven at 85 °C for 45 min (Figure 2a). The cured PDMS devices were peeled off from the molds, followed by hole punching using a biopsy punch and cleaning with alcohol and DI water to obtain the blood and gas layers (Figure 2b). Next, the degassed PDMS solution was spin-coated on a 6-inch silane-treated silicon wafer at a speed of 3000 rpm for 60 s, followed by curing at 85 °C for 45 min to form a PDMS membrane with a thickness of approximately 20 μm (Figure 2c). Finally, the PDMS-made blood layer, permeable membrane and gas layer were sequentially treated via air plasma activation (PLUTO-T, Plutovac, Shanghai, China) and were brought in contact (Figure 2d,e). Cross-shaped markers fabricated on the blood and gas layers ensure precise alignment of layers during bonding. The assembled PDMS device was stored at 85 °C overnight to complete the bonding and form the complete MO device. Due to the inherent limitations of fabrication method, the cross-sections are rectangular, which deviates from the physiological conditions. The non-circular channels may exhibit altered velocity profiles, localized flow recirculation, and non-uniform mass transport compared to physiologically relevant round lumens. This is acknowledged as a limitation of the current approach.

The fabricated single-layer MO device has an overall size of 13 cm × 13 cm (Figure 2f). The front view of the oxygenator, observed under an optical microscope (MF53-N, Mshot, Guangzhou, China), shows well-aligned micropillars of the gas layer and one clear secondary channel and two capillary channels of the blood layer (Figure 2g). A cross-sectional image obtained by scanning electron microscope (GEMINI300, ZEISS, Oberkochen, Germany) shows that the PDMS permeable membrane (~20 μm thickness) is firmly bonded to the structural surface of the blood layer (upper) and the gas layer (lower) without collapse, ensuring effective gas exchange (Figure 2h,i). To verify sealing performance of the assembled MO, physiological saline was perfused into both blood and gas layers at a flow rate of 5–20 mL/min, with no leakage observed.

### 2.5. Hemocompatibility Assessment

The hemocompatibility of the fabricated MO devices was evaluated by examining plasma free hemoglobin (fHb), coagulation process, and platelets. In vitro circulation experiments were performed using 20 mL of bovine blood, at flow rates of 5 mL/min and 8 mL/min in a peristaltic pump-only circuit and a pump–MO circuit (Figure S1). Notably, the flow rate of 5 mL/min per layer was a target for efficient oxygenation, while 8 mL/min per layer was expected to meet the requirement of flow rates (64 mL/min) for 1280 g preterm infants via multilayer stacking. Blood samples were collected at different circulation time points. Plasma was separated by centrifugation (250 g, 10 min), then treated and incubated with a hemoglobin assay kit. The fHb was calculated by measuring the absorbance of the plasma at 37 °C using a UV spectrophotometer (U-3900H, Hitachi, Tokyo, Japan), reflecting the degree of hemolysis caused by mechanical shear force during circulation. Coagulation cascade reaction was triggered by kaolin reagents (extrinsic pathway) and CaCl_2_ (recalcification), and measured at 37 °C using a thromboelastography analyzer (TEG 5000, Haemonetics, Boston, MA, USA), to investigate whether blood was activated during circulation. Platelet count and cell diameter were measured using a cell counter (Countstar IC 1000, Countstar BioTech, Shanghai, China) to evaluate blood cell integrity. Citrated (3.2%) bovine blood was used for all hemocompatibility experiments. The total hemoglobin concentration of the blood used in this experiment was 11.1 g/dL.

### 2.6. Experimental Setup for Blood Oxygenation

The schematic diagram of MO-based system for blood oxygenation is shown in Figure S2. The inlets and outlets on both the blood and gas layers were connected to silicone tubing via stainless steel tubes. A peristaltic pump (LM60A, Runze Fluid control, Nanjing, China) was used to perfuse bovine blood through the silicone tubing into the blood layer of the MO. Two pressure sensors (Prinzen, Shanghai, China) were separately set at the inlet and outlet of the blood layer, providing real-time pressure drop across the oxygenator. Simultaneously, dry oxygen or air was supplied to the gas layer at a flow rate of 20 mL/min through a flow controller (MFC300, Aitoly, Suzhou, China). A heating water bath (JoanLab, Huzhou, China) was used to maintain the blood at 37 °C. Oxygenated blood was collected at the outlet of the MO device and was analyzed using a blood gas analyzer (RAPIDPoint^®^ 500e, Siemens, Tarrytown, NY, USA). The blood gas parameters, such as oxygen saturation (SO_2_), partial pressure of oxygen (PO_2_), total hemoglobin (tHb), and hematocrit (Hct), can be obtained. To avoid the influence of ambient oxygen on the oxygenation measurements, blood samples collected were performed for blood gas analysis within 10 min. The MO-based circuit was flushed with physiological saline at a flow rate of 5 mL/min for 5 min prior to blood perfusion. Before each oxygenation experiment on the MO devices, the bovine blood was deoxygenated to a venous condition (SO_2_ = 65% ± 5%, PCO_2_ = 45–55 mmHg, base excess 0 ± 3 mmol/L, glucose concentration 4–8 mmol/L) by passing carbon dioxide and nitrogen gas through a hollow fiber-based oxygenator (OX-200, STMed, Suzhou, China), as shown in Figure S3. Heparinized bovine blood (4 U/mL) was used for all blood oxygenation experiments to avoid dilution-induced bias in blood gas measurements.

### 2.7. Performance Evaluation

We employed vol% oxygen transfer to evaluate the oxygenation performance of the microfluidic oxygenators. It comprises the oxygen bound to hemoglobin and the oxygen dissolved in the plasma, as described by [49]:

(5) $$∆V_{O2}=1.35\times tHb\times ∆{SO}_{2}+0.0031\times ∆{PO}_{2}$$

where tHb represents the total hemoglobin content in the blood, ΔSO_2_ denotes the change in oxygen saturation, and ΔPO_2_ indicates the change in partial pressure of oxygen. When the tHb is 12.0 g/dL and the SO_2_ rises from 65% to 95%, the vol% oxygen transfer calculated by Equation (5) is 4.95%, indicating an oxygen uptake of 4.95 mL per deciliter of blood [50]. The tHb of the bovine blood used in our experiments was 11.1 g/dL. Accordingly, when the SO_2_ increases from 65% to 95%, the corresponding vol% oxygen transfer is adjusted to 4.59%.

### 2.8. Materials

PDMS (Sylgard 184, Dow Corning, Midland, MI, USA) base and curing agent were purchased from Suzhou Cchip Scientific Instrument Co., Ltd. (Suzhou, China). Silicone rubber tubing and Luer connectors were obtained from Nanjing Runze Fluid Control Equipment Co., Ltd. (Nanjing, China). A free hemoglobin assay kit was purchased from Beijing Solarbio Science and Technology Co., Ltd. (Beijing, China). Solid heparin-treated bovine blood (4 U/mL) and citrated (3.2%) bovine blood were acquired from Shanghai Yuchun Biology Science and Technology Co., Ltd. (Shanghai, China). Carbon dioxide (purity 99.9%), oxygen (purity 99.9%), and air (purity 99.9%) were supplied by Jinhong Gas Co., Ltd. (Suzhou, China).

## 3. Results and Discussion

### 3.1. 3D Modeling of the Blood Layer

**Figure 3:**
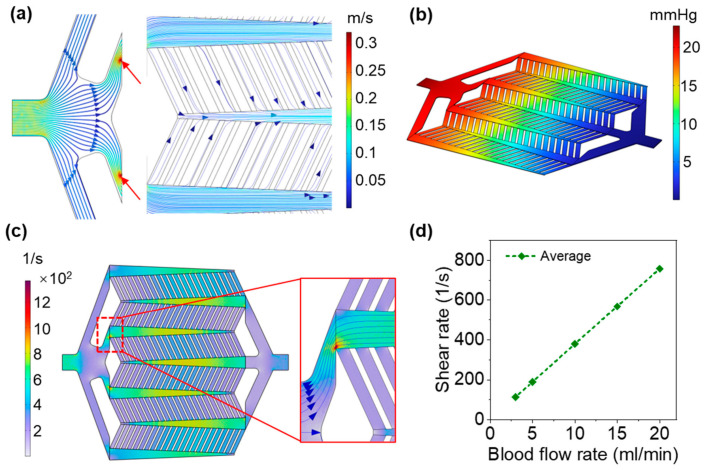
3D simulation results of blood layer of the MO. (**a**) Simulated velocity streamlines in the MO at a blood flow rate of 5 mL/min. (**b**) Simulated pressure drop at a blood flow rate of 5 mL/min per layer. The arrows indicate the regions of maximum flow velocity. (**c**) Distribution of shear rate and localized high-shear regions at a blood flow rate of 5 mL/min. The red square marks the locations of the maximum shear rate. (**d**) Simulated average wall shear rate (1/s) at blood flow rates from 3 to 20 mL/min for a single layer.

Based on the structure design above, the entire blood channels were simulated using computational fluid dynamics (CFD) in COMSOL Multiphysics 6.0 (Figure 3). The blood flow rate at the inlet was set to 5 mL/min according to the target. Velocity streamlines within multi-level channels are uniform, with the maximum velocity (~0.32 m/s) occurring at the junction between the primary and secondary channels (Figure 3a). The pressure drop across the entire device is 22.8 mmHg (Figure 3b), which is lower than the MAPD of 35–45 mmHg in preterm infants weighing 500–700 g [29,47], indicating that pump-free circulation may be achievable when the external circuit pressure drop remains below 22.2 mmHg.

In human blood vessels, typical shear rates are approximately 300 s^−1^ in the femoral artery, 1500 s^−1^ in small arterioles, and up to 2800 s^−1^ near the capillaries [51,52]. The simulated maximum blood shear rate in the designed MO device is 1300 s^−1^, also located at the junction between the primary and secondary channels. (Figure 3c). Although locally elevated, the average shear rate across the entire blood channel remains within a safe range of 100–800 s^−1^ at flow rates of 3–20 mL/min (Figure 3d). Additionally, previous studies indicate that such transient, localized high-shear rates induce minimal blood damage [53]. Although this claim has been met with dissenting opinions [54], subsequent hemocompatibility tests will yield more definitive results.

### 3.2. Hemocompatibility Tests

**Figure 4:**
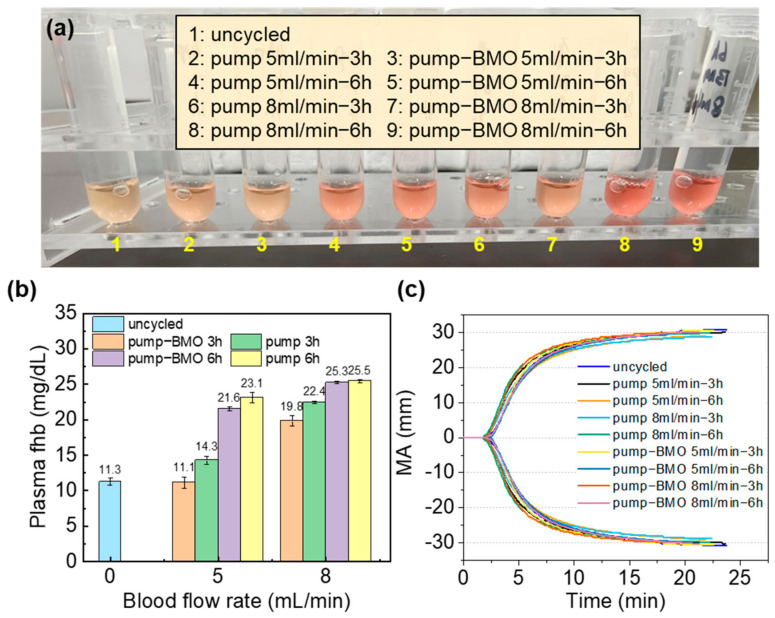
Hemocompatibility testing. (**a**) Plasma samples centrifuged from citrated bovine blood circulating through the pump-only circuit and pump–MO circuit at flow rates of 5 and 8 mL/min for 3 and 6 h, respectively. (**b**) Measured plasma fHb levels of the samples. Data are means ± SD and *n* = 3. (**c**) Thromboelastography kaolin test of the circulating citrated bovine blood.

Following the method described in Section 2.5 (Hemocompatibility assessment), centrifuged plasma samples were obtained from citrated blood circulating through the pump-only and pump–MO circuits at flow rates of 5 mL/min and 8 mL/min for 3 h and 6 h, respectively (Figure 4a). Intuitively, the plasma from the pump-only circuit was slightly darker in color than that from the pump–MO circuit. The measured fHb levels are presented in Figure 4b. For initial uncycled blood samples, the fHb level was 11.3 mg/dL. Although this initial fHb level exceeded the normal range (5 mg/dL), the relative changes in fHb of the circulating blood could still be compared. At a flow rate of 5 mL/min, the fHb levels in the pump–MO circuit were 11.1 mg/dL after 3 h and 21.6 mg/dL after 6 h, whereas those in the pump-only circuit were slightly higher (14.3 mg/dL at 3 h and 23.1 mg/dL at 6 h). Similarly, at 8 mL/min, the fHb levels in the pump–MO circuit were also lower than those in the pump-only circuit after 3 and 6 h of circulation. Interestingly, for the same circulation duration, the difference in fHb levels between the two circuits was smaller at the higher flow rate of 8 mL/min compared to that at 5 mL/min. For the maximum measured fHb of 25.5 mg/dL (pump only, after 6 h at 8 mL/min), the hemolysis percentage was calculated to be 0.23% according to ASTM F756-17 (the ratio of fHb to tHb), which is well below the 2% threshold for a non-hemolytic classification [55]. These results indicate that the peristaltic pump is the primary factor causing blood damage, while the MO in the circuit is expected to mitigate the pulsatile effect of the peristaltic pump on blood, as previously reported membrane oxygenators [56].

Furthermore, thromboelastography assay was performed on the circulating citrated blood samples to evaluate clotting activation induced by the MO device (Figure 4c). The reaction time (R) and amplitude (MA) represent the coagulation initiation and clot strength, respectively, both of which are influenced by platelets and their released substances. When platelet function is enhanced, R will shorten and MA will increase. As shown in Figure 4c, no significant changes were observed among different circulating blood samples, indicating that platelets were not activated by shear forces during circulation. Detailed coagulation parameters, platelet concentration, and blood cell diameters are summarized in Tables S2 and S3. These experiments demonstrate that the MO device developed in this work exhibits good hemocompatibility during extracorporeal circulation.

### 3.3. Pressure Drop Testing of 8-Layer MOs

**Figure 5:**
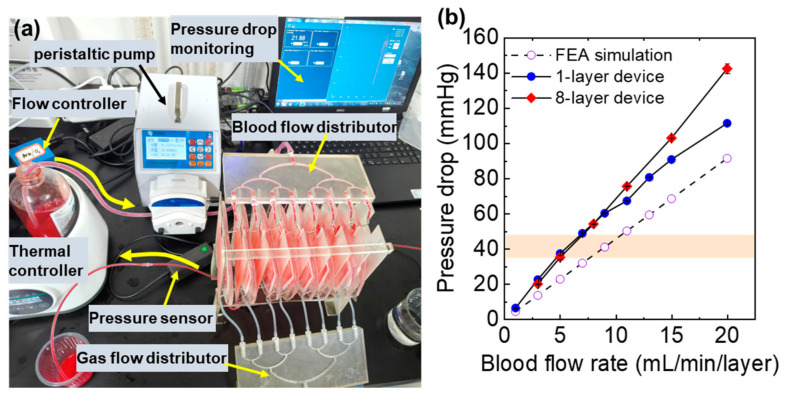
Measurements of pressure drops of the 8-layer MO using heparinized bovine blood. (**a**) Experimental setup. (**b**) Measured pressure drop for one-layer and eight-layer MO devices at various blood flow rates per layer, and the simulated pressure data (from CFD model in Section 3.1) of one-layer device. Data are means ± SD and *n* = 3.

To accommodate preterm infants with different body weights (i.e., different blood flow rate and oxygen uptake), we made a scale-up for the single-layer MO to an eight-layer stack using flow distributors. Figure 5a shows an experimental setup for measuring the pressure drop of the eight-layer device. The flow distributors were produced by 3D printing, with their internal channels branching multiple times to form eight ports. Bovine blood was delivered into eight MO devices through the distributor, and flowed out from another distributor at the outlet of MO. The pressure sensors were placed at the upstream and downstream of the inlet and outlet distributors, respectively. Other configurations are the same as those described in Section 2.6 (experimental setup for blood oxygenation). The uniformity of flow rates across eight MOs is essential for achieving optimal oxygenation, which can be verified using a simple volume quantification method. The results indicate a relatively uniform flow distribution among the eight devices (as shown in Figure S4). A recorded video is provided in the Supplementary Information (SI).

Figure 5b shows the pressure drop for one-layer and eight-layer stacked MO at various blood flow rates from 1 to 20 mL/min. It shows that the pressure drops across the MO devices linearly increased with flow rates. At flow rate of 1 to 10 mL/min per layer, the total pressure drops for the one-layer and the eight-layer MO device are nearly equivalent, demonstrating that the parallel configuration does not significantly increase flow resistance, also proving that the flow distribution in parallel eight layers is uniform. When the flow rate of eight-layer device exceeds 120 mL/min (15 mL/min per layer), the pressure drops are 10–25 mmHg higher than those of the one-layer device. This may be attributed to increased flow resistance from the external tubing. For preterm infants with birth weights ranging from 500 g to 700 g (with a typical blood flow rate of 50 mL/min/kg), the MAPD gradually increases from 35 to 45 mmHg between 3 and 96 h after birth, depending on both birth weight and postnatal age, and this value continues to rise with increasing postnatal age [29,47]. Considering that a pressure drop of 10 mmHg needs to be reserved for the external circuit, the pressure drop of the MO device should be in a range of 25 to 35 mmHg. Therefore, when the blood flow rate per layer is 3–5 mL/min (equivalent to a total blood flow rate of 24–40 mL/min), pumpless extracorporeal circulation support can be provided for preterm infants with a birth weight of 500–700 g.

### 3.4. Blood Oxygenation Performance of the MO

#### 3.4.1. Optimization of Experimental Conditions

**Figure 6:**
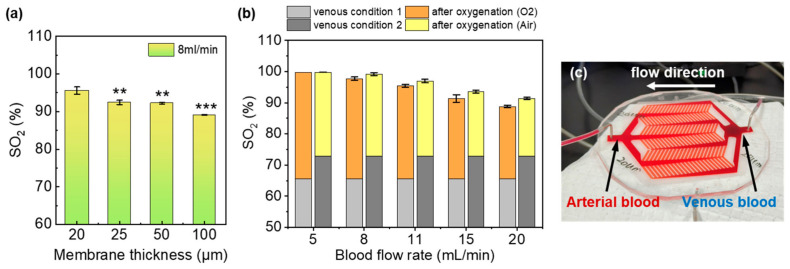
Optimization of experimental conditions for MO using heparinized bovine blood. (**a**) Effect of PDMS membrane thickness on oxygenation efficiency. Initial SO_2_ is 59%. One way ANOVA followed by Tukey test, baseline = 20 μm, *p* ≤ 0.05 indicates a statistically significant difference. ** represents *p* ≤ 0.01, and *** represents *p* ≤ 0.001. (**b**) Impact of sweep gas composition on the blood oxygenation. The initial venous condition of blood differed between the two groups. (**c**) A photograph of the blood oxygenation progress in a single-layer oxygenator. Data are means ± SD and *n* = 3.

The thickness of gas exchange membrane is a critical parameter determining oxygenation performance in oxygenators. To investigate this effect, heparinized bovine blood (initial SO_2_: 59%) was perfused sequentially through MO devices equipped with PDMS membranes of 20, 25, 50, and 100 μm thickness at a flow rate of 8 mL/min. Blood samples collected from the outlet were analyzed using the blood gas analyzer. Results show that SO_2_ gradually decreases as membrane thickness increases (Figure 6a). The device with a 20 μm PDMS membrane achieved an SO_2_ >95% at 8 mL/min, while those with thicker membranes failed to reach adequate oxygenation level. Tukey test comparisons revealed that the 20 µm group (95.57 ± 0.99%) was significantly higher than the 25 µm, 50 µm, and 100 µm groups (all *p* < 0.001). Although thinner membranes can achieve higher gas-exchange efficiency, reduced membrane thickness compromises mechanical strength, making the membrane difficult to withstand lateral pressure under high flow rates, which may lead to deformation or rupture. Based on these results, a PDMS membrane thickness of 20 μm was selected for all MO devices.

To assess the influence of sweep gas composition on oxygenation, we compared the blood oxygenation under pure oxygen and air supply in the MO device. Both gas supplies achieved SO_2_ of over 95% when the flow rate was below 11 mL/min (Figure 6b). When pure oxygen was used, the change in SO_2_ of blood at the outlet of blood layer was approximately 5.7% higher than that under air supply at blood flow rates of 5, 8, 11, 15, and 20 mL/min. Figure 6c presents a photograph of blood transition from venous to arterial in the single-layer MO, with a blood flow rate of 5 mL/min and oxygen flow rate of 20 mL/min.

#### 3.4.2. Oxygenation Performance of Single-Layer MO

**Figure 7:**
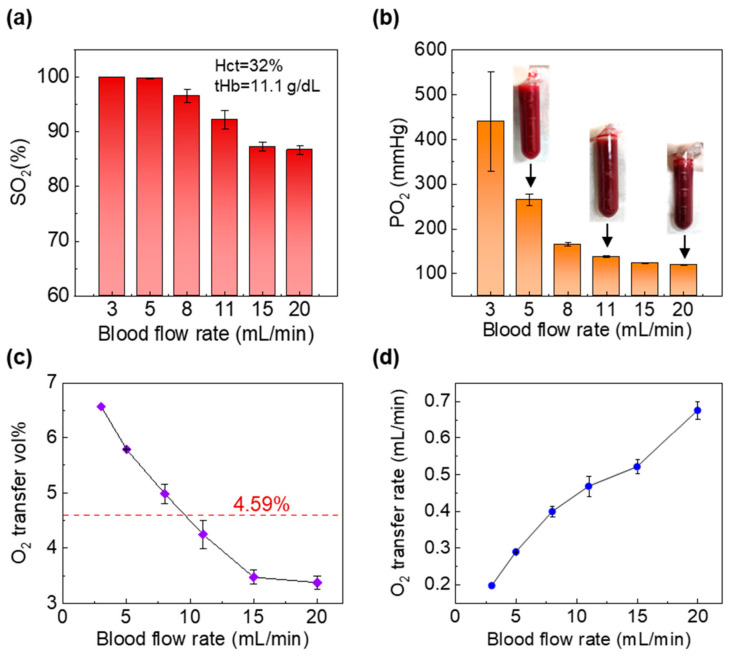
In vitro blood oxygenation results for MO using heparinized bovine blood. Value of (**a**) SO_2_ and (**b**) PO_2_ measured at different blood flow rates. The initial SO_2_ is 64.7%. Inset shows oxygenated blood samples. (**c**) Normalized vol% oxygen transfer percentage and (**d**) normalized oxygen transfer rate derived from (**a**,**b**). Data are means ± SD and *n* = 3.

After optimizing the experimental conditions, we conducted performance verification of the MO device using heparinized bovine blood. Heparinized bovine blood was deoxygenated to venous conditions (as shown in Figure S3), with SO_2_ of 64.7%, Hct of 32%, and tHb of 11.1 g/dL. Figure 7a,b present the measured SO_2_ and PO_2_ of oxygenated blood passing through a single-layer MO at flow rates from 3 to 20 mL/min (3, 5, 8, 11, 15, and 20 mL/min), with an oxygen supply rate of 20 mL/min. The device achieved an SO_2_ of 99.7% at a blood flow rate of 5 mL/min and 96.5% at 8 mL/min. As the blood flow rate increased, both the SO_2_ and PO_2_ gradually decreased. Nevertheless, the device still maintained an SO_2_ of 85% at 20 mL/min. The inset in Figure 7b shows photographs of the oxygenated blood at flow rates of 5, 11, and 20 mL/min.

Figure 7c exhibits the vol% oxygen transfer calculated from the data in Figure 7a,b and Equation (5). At flow rates ≤ 8 mL/min, the single-layer MO device achieved a vol% oxygen transfer of 4.98%, which is equivalent to 5.24% when the tHb content is 12.0 g/dL. Notably, these vol% oxygen transfer results also indirectly indicate a maximal working flow rate of about 64 mL/min for the eight-layer MO device (corresponding to 8 mL/min per layer). The value was derived by scaling up the single-layer device’s performance and aligns with the flow uniformity performance verified earlier. Consequently, our eight-layer MO device is capable of supporting extracorporeal oxygenation for infants with a maximum birth weight of 1280 g (corresponding to a gestational age of approximately 28–29 weeks). At a flow rate of 20 mL/min, the vol% oxygen transfer was approximately 3.38%. Furthermore, the oxygen transfer rate (i.e., the volume of oxygen transferred from the gas layer to the blood layer per unit time) can be obtained by multiplying the vol% oxygen transfer by the blood flow rate [49], and the oxygen transfer rate per unit area can be obtained by dividing the oxygen transfer rate by the total oxygenation area. Figure 7d illustrates the relationship between the oxygen transfer rate and the blood flow rate. At the nominal blood flow rate of 8 mL/min, the measured vol% oxygen transfer was 4.98%, resulting in the oxygen transfer rate of 0.398 mL O_2_/min. Additionally, the device achieved an oxygen transfer rate per unit area of 95 mL/min/m^2^, and the normalized oxygen transfer exceeds 52.4 mL O_2_/L blood (equivalent to a tHb content of 12 g/dL), which is comparable to clinical oxygenators and confirms its potential for practical application.

However, due to the uneven gas flow distribution caused by the current gas distributor, oxygenation experiments of the eight-layer device could not be reliably performed in this work. In future work, we will redesign and optimize the gas distributor to ensure uniform gas flow across all layers. Importantly, the priming volume for the eight-layer device is 5.6 mL, and the total priming volume including external circuit (two manifolds, tubing and connectors) is 10.2 mL, which exceeds the conventional safety limit of 10% of the total blood volume in extremely preterm infants undergoing extracorporeal circulation. Even so, this priming volume is still significantly lower than that of hollow fiber oxygenators, and can effectively reduce high-priming-volume-related complications. For such low-body-weight infants, maternal blood priming can be adopted to maintain stable circulatory volume without additional blood load on neonates. Compared to the existing microfluidic oxygenators (as shown in Table S4), our MO represents a notable advance in balancing hemocompatibility, oxygenation efficiency, operational flow rates, and physiology-mimicking structures. Its scalable merit allows precise matching to the varying blood volumes and flow requirements of preterm infants of different gestational ages.

## 4. Conclusions

This study presents a physiology-mimicking microfluidic oxygenator for preterm infants, addressing the urgent need for suitable extracorporeal respiratory support. The device features a multi-level channel structure mimicking human vasculature to enhance hemocompatibility, with a priming volume of 0.72 mL for a single-layer device. Hemolysis tests, coagulation measurements, and platelet assessments of blood circulating through the device for 6 h demonstrated favorable hemocompatibility. The pressure drop across the device suggests potential for pumpless circulation for 700 g premature neonates. Oxygenation experiments showed oxygen saturation increased from 64.7% to 96.5% and oxygen transfer rate reached 95 mL/min/m^2^ under 8 mL/min, which might meet the blood flow rates of neonates with body weight below 1280 g by multilayer stacking. These advancements hold promise for providing a minimally invasive, highly safe, and customizable extracorporeal respiratory support solution for premature infants. This study still has limitations that oxygenation performance verification of multilayer device has not been completed. Future work will further optimize the device structure and explore in vitro application in animal models.

## Data Availability

The data presented in this article are available on request from the corresponding authors.

## Supplementary Materials

The following supporting information can be downloaded at https://www.mdpi.com/article/10.3390/mi17060745/s1, Figure S1: Experimental setup for hemolysis tests: pump-only circuit and pump–MO circuit; Figure S2: Schematic of the blood oxygenation system. The setup includes a peristaltic pump, a heating circulator, a flow controller, an oxygen cylinder, a sampling port, pressure sensors, and the MO; Figure S3: Experimental setup of deoxygenation process of bovine blood by a hollow fiber-based oxygenator (OX-200, STMed, Suzhou, China); Figure S4: Flow distribution testing for 8-layer MO. Physiological saline was infused into the inlet of the distributor at a total flow rate of 64 mL/min, and collected from the outlet of each MO device at 0, 20, 40, and 60 s. The volumes of physiological saline in eight graduated cylinders were 8 ± 0.5 mL; Table S1: Material properties of a PDMS membrane; Table S2: Hemocompatibility assessment of blood after circulation through pump-only circuit and pump–MO circuit. Effects on coagulation parameters (R: reaction time, MA: maximum amplitude), platelet concentration (PC), and cell diameter (D_cell_) under different flow rates (V_blood_, mL/min) and circulation times (T, h). “-” indicates not measured. Values are presented as mean ± SD (*n* = 3); Table S3: *p*-values for statistical comparisons between the pump-MO circuit and the pump-only circuit under the same flow rate and circulation time. The raw data used to calculate the *p*-values were derived from Table S2. Unpaired t tests were used for each pair of conditions (pump-only and pump–MO). A *p* value ≤ 0.05 (*) indicates a statistically significant difference; Table S4: Performance comparison to the previously reported microfluidic blood oxygenators. Parameters include membrane thickness (H), priming volume (P_Vol_), pressure drop (Δ*P*), oxygenation area, vol% oxygen transfer, the variation of SO_2_ from venous to arterial blood, and so on; Video S1: flow uniformity test_8layer_8mlmin [57,58,59].
