# Supplementary material for: Physiology-Mimicking Microfluidic Oxygenator with Good Hemocompatibility for In Vitro Respiratory Support of Preterm Infants
Source: Micromachines (Basel). 2026 Jun 20;17(6):745. doi: 10.3390/mi17060745 (PMC13302908; doi:10.3390/mi17060745)
Supplement: Supplementary file 1 [file micromachines-17-00745-s001.zip › micromachines-4306780-supplementary.pdf]

micromachines

Supplementary Materials

Physiology-mimicking Microfluidic Oxygenator with Good Hemocompatibility for In Vitro Respiratory Support of Preterm Infants

Yu Tao, Yao Lu, Weijun Zeng, Donggen Xiao and Haixuan Sun

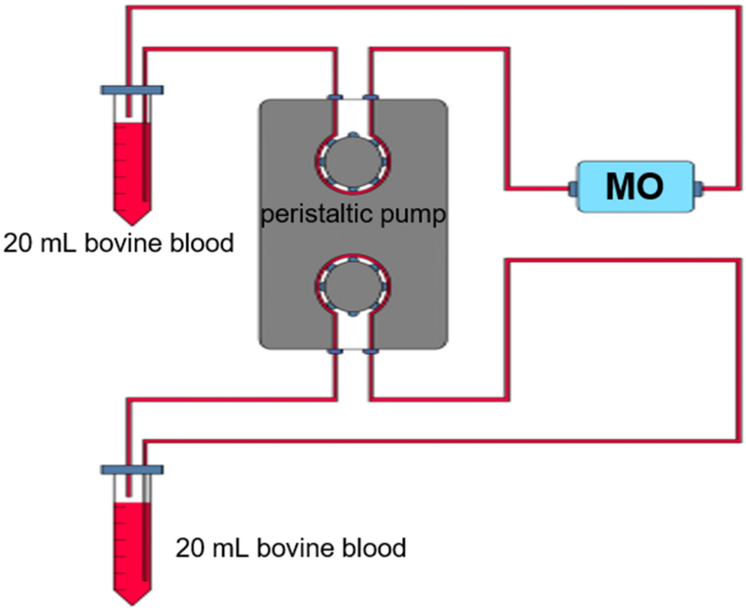

Figure S1. Experimental setup for hemolysis tests: pump-only circuit and pump-MO circuit.

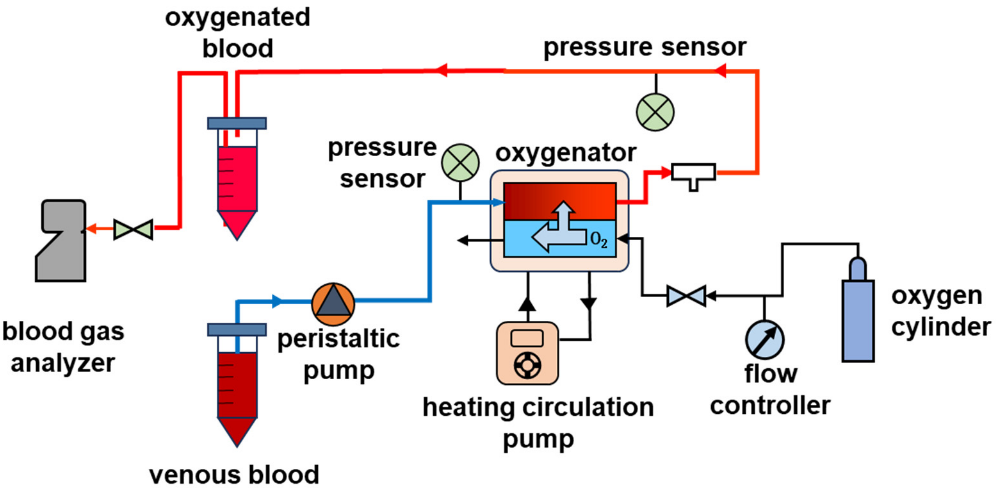

Figure S2. Schematic of the blood oxygenation system. The setup includes a peristaltic pump, a heating circulator, a flow controller, an oxygen cylinder, a sampling port, pressure sensors, and the MO.

 https://doi.org/10.3390/mi17060745

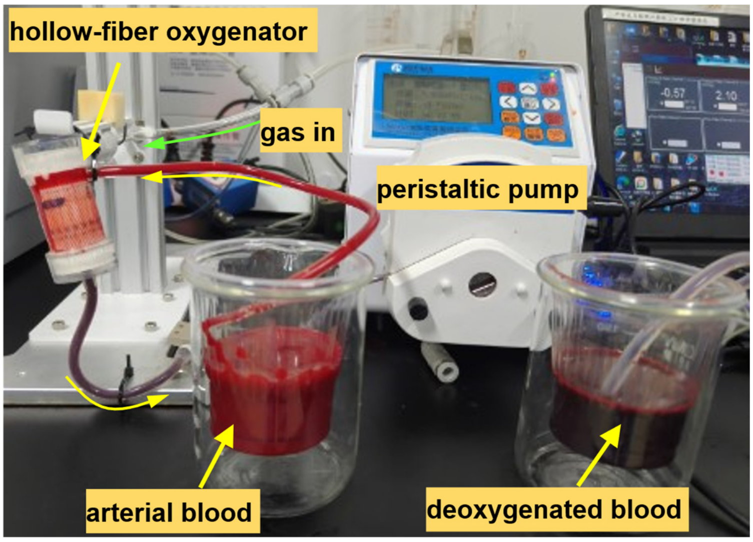

Figure S3. Experimental setup of deoxygenation process of bovine blood by a hollow fiber-based oxygenator (OX-200, STMed, Suzhou, China).

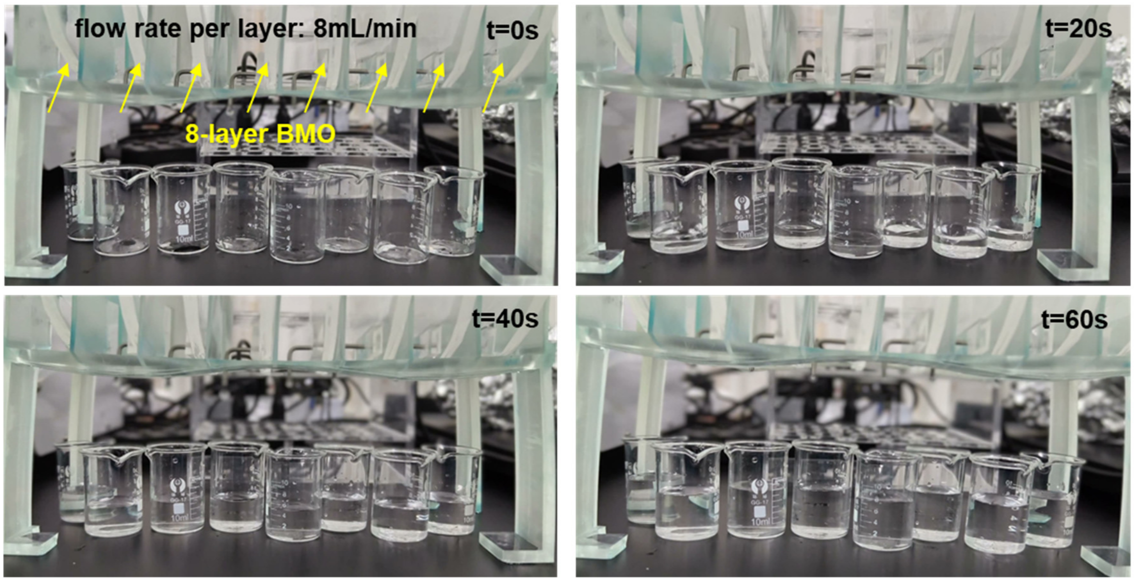

Figure S4. Flow distribution testing for 8-layer MO. Physiological saline was infused into the inlet of the distributor at a total flow rate of 64 mL/min, and collected from the outlet of each MO device at 0, 20, 40, and 60 s. The volumes of physiological saline in eight graduated cylinders were 8±0.5 mL.

https://doi.org/10.3390/mi17060745

Table S1. Material properties of a PDMS membrane.

Membrane Thickness	Oxygen Transmission Rate $P_{M,O2}$	Tensile Strength	Elastic Modulus	Tear Strength	Surface Adhesion
20 μm	4.35×10^{-9} mL O2/cm/s/mmHg	4 MPa	1.7 MPa	7 KN/m	low
25 μm	3.48×10^{-9} mL O2/cm/s/mmHg	5.5 MPa	2.3 MPa	20 KN/m	moderate
50 μm	1.74×10^{-9} mL O2/cm/s/mmHg	5.5 MPa	2.3 MPa	20 KN/m	moderate
100 μm	0.87×10^{-9} mL O2/cm/s/mmHg	5.5 MPa	2.3 MPa	20 KN/m	moderate

Table S2. Hemocompatibility assessment of blood after circulation through pump-only circuit and pump–MO circuit. Effects on coagulation parameters (R: reaction time, MA: maximum amplitude), platelet concentration (PC), and cell diameter (D_{cell}) under different flow rates (V_{blood}, mL/min) and circulation times (T, h). "-" indicates not measured. Values are presented as mean ± SD (n = 3).

Circulation circuit	V_{blood} (mL/min)	R (min)	MA (mm)	PC ($\times10^7$ /mL)	D_{cell} (μm)
uncycled	-	2.50±0	60.90±1.13	4.53±0.72	6.11±0.07
Pump	5	2.33±0.06	57.40±2.46	-	-
Pump–MO	5	2.27±0.31	63.20±4.73	-	-
Pump	5	2.40±0.10	56.93±0.64	4.41±0.09	5.95±0.23
Pump–MO	5	2.53±0.06	58.83±0.86	4.45±0.15	6.15±0.04
Pump	8	2.67±0.32	53.77±3.51	-	-
Pump–MO	8	2.40±0.44	58.23±2.89	-	-
Pump	8	2.10±0	58.63±1.59	4.43±0.18	6.08±0.06
Pump–MO	8	2.27±0.23	59.93±1.50	4.73±1.09	6.15±0.17

Table S3. *p*-values for statistical comparisons between the pump–MO circuit and the pump-only circuit under the same flow rate and circulation time. The raw data used to calculate the *P*-values were derived from Table S2. Unpaired t-tests were used for each pair of conditions (pump-only and pump–MO). A *p*-value ≤ 0.05(*) indicates a statistically significant difference.

V_{blood} / T	*p*–(R)	*p*–(MA)	*p*–(PC)	*p*–(D_{cell})
5 mL/min-3 h	0.735	0.155	-	-
5 mL/min-6 h	0.125	0.042*	0.709	0.212
8 mL/min-3 h	0.422	0.164	-	-
8 mL/min-6 h	0.602	0.363	0.667	0.539

The detailed performance parameters compared to the reported microfluidic oxygenators are summarized in Table S4. The oxygenator designed by Dabaghi et al. achieved an increase in oxygen saturation from approximately 65% to 88% at a flow rate of 3.75 mL/min for a single-layer device with air as sweep gas [1]. When pure oxygen was used, it can increase SO_2 from about 62% to 95% at a flow rate of 6.25 mL/min per layer. Despite the significant improvement in oxygen saturation, the blood flow rate in the single-layer device remains low, and the oxygen transfer per unit area is limited. Gimbel et al. developed a 14-layer stacked microfluidic oxygenator with a small priming volume of 0.04 mL per layer, achieving an oxygen transfer efficiency of 304 mL/min/m^2, while this design yields a pressure drop greater than 100 mmHg across the device [2]. Lachaux et al. demonstrated a higher blood flow rate within a single-layer device and reached an oxygen transfer rate of 318 mL/min/m^2; however, single-layer device limited the final oxygen saturation to below 95% at 10 mL/min [3]. Neda Saraei et al. reported a vol% oxygen transfer of 4.98% with pure oxygen as the sweep gas, through the oxygen transfer per unit area was relatively moderate [4]. In a more recent study, Blauvelt et al. fabricated presented a silicon membrane-based microfluidic oxygenator that operates with minimal anticoagulation (activated clotting time: 120–180 s), which delivered a vol% oxygen transfer of 1.51% [5]. Moreover, the device adopted a blood channel height of 500 μm, leading to a priming volume of 1.5 mL per layer, which is considerably larger than that of our device. Though

the listed microfluidic oxygenators for comparative evaluation are insufficient, our device delivers improved comprehensive performance by balancing hemocompatibility, oxygenation efficiency, operational flow rates, and physiology-mimicking structures.

Table S4. Performance comparison to the previously reported microfluidic blood oxygenators. Parameters include membrane thickness (H), priming volume (PVol), pressure drop (ΔP), oxygenation area, vol% oxygen transfer, the variation of SO_2 from venous to arterial blood, and so on.

Device	H (μm)	Max Blood Flow Rate (mL/min)	PVol (mL)	Sweep Gas	ΔP (mmHg)	Vol% O_2 Transfer	SO_2 Variation	Effective Surface Area (cm^2)	Max O_2 Transfer (mL/min)	O_2 Transfer Efficiency ($mL/min/m^2$)
current	80	8	0.72	O_2	54	4.98%	31.8%	42	0.398	95
Ref. [1]	180	3.75	0.78	Air	34	3.04%	23%	37.5	0.114	30
		6.25		O_2	50	5.71%	33%	37.5	0.357	95
Ref. [2]	50	1.96	0.04	O_2	>100	4.95%	-	3.2	0.097	304
Ref. [3]	105	10	0.273	O_2	67	3.4%	25%	10.7	0.34	318
Ref. [4]	105	10	1.56	Air	40	4.44%	22.2%	150	0.4425	30
	105	18.75		O_2	69	4.98%	21.4%	150	0.935	62
Ref. [5]	500	10	1.5	O_2	28	1.51%	-	15.4	0.151	98

Reference

1. Dabaghi, M.; Rochow, N.; Saraei, N.; Fusch, G.; Monkman, S.; Da, K.; Shahin-Shamsabadi, A.; Brash, J.L.; Predescu, D.; Delaney, K.; et al. A Pumpless Microfluidic Neonatal Lung Assist Device for Support of Preterm Neonates in Respiratory Distress. *Adv. Sci.* **2020**, *7*, 2001860. https://doi.org/10.1002/advs.202001860.
2. Gimbel, A.A.; Hsiao, J.C.; Kim, E.S.; Lewis, D.J.; Risoleo, T.F.; Urban, J.N.; Borenstein, J.T. A High Gas Transfer Efficiency Microfluidic Oxygenator for Extracorporeal Respiratory Assist Applications in Critical Care Medicine. *Artif. Organs* **2021**, *45*, E247–E264. https://doi.org/10.1111/aor.13935.
3. Lachaux, J.; Hwang, G.; Arouche, N.; Naserian, S.; Harouri, A.; Lotito, V.; Casari, C.; Lok, T.; Menager, J.B.; Issard, J.; et al. A compact integrated microfluidic oxygenator with high gas exchange efficiency and compatibility for long-lasting endothelialization. *Lab Chip* **2021**, *21*, 4791–4804.
4. Saraei, N.; Dabaghi, M.; Fusch, G.; Rochow, N.; Fusch, C.; Selvaganapathy, P.R. Scaled-up Microfluidic Lung Assist Device for Artificial Placenta Application with High Gas Exchange Capacity. *ACS Biomater. Sci. Eng.* **2024**, *10*, 4612–4625.
5. Blauvelt, D.G.; Higgins, N.C.; Hesek, A.; De, B.N.; Wright, N.; Nithianandam, P.; Blaha, C.; Moyer, J.; Chui, B.W.; Baltazar, F.J.; et al. A silicon membrane microfluidic oxygenator for use as an artificial placenta with minimal anticoagulation. *Bioeng. Transl. Med.* **2025**, *10*, e70037.

Disclaimer/Publisher's Note: The statements, opinions and data contained in all publications are solely those of the individual author(s) and contributor(s) and not of MDPI and/or the editor(s). MDPI and/or the editor(s) disclaim responsibility for any injury to people or property resulting from any ideas, methods, instructions or products referred to in the content.
